# Attention-deficit hyperactivity disorder symptoms and quality of life in female patients with fibromyalgia

**DOI:** 10.3906/sag-2010-29

**Published:** 2021-08-30

**Authors:** Gözde TÜRKOĞLU, Yavuz SELVİ

**Affiliations:** 1 Department of Physical Medicine and Rehabilitation, University of Health Sciences, Konya Training and Research Hospital, Konya Turkey; 2 Department of Psychiatry, Faculty of Medicine, Selçuk University, Konya Turkey

**Keywords:** Adult attention deficit hyperactivity disorder, fibromyalgia, quality of life

## Abstract

**Background/aim:**

The present study aimed to determine the association between attention-deficit hyperactivity disorder (ADHD) symptoms severity, fibromyalgia (FM) severity, and QoL.

**Materials and methods:**

While the FM group consisted of 113 (74%) patients, the control group consisted of 40 (26%) individuals. FM symptom severity, ADHD symptom severity, and QoL were evaluated using the fibromyalgia impact questionnaire (FIQ), adult ADHD self-report scale (ASRS), and World Health Organization quality of life scale-brief version (WHOQOL-BREF), respectively.

**Results:**

It was found that the FM group had significantly higher scores on the ASRS than the control group (p < 0.05). There was a significant difference in FIQ scores and three WHOQOL-BREF domain scores between the FM alone and comorbid FM/high probability of ADHD groups (p < 0.05). We found a negative correlation between ASRS total scores and all other scale scores (except for the social relationships domain score of the WHOQOL-BREF) and a positive correlation between ASRS total scores and FIQ scores in FM patients. ADHD scores would mediate the relationship between depression severity and QoL.

**Conclusions:**

Our findings indicated that the presence of ADHD symptoms was related to greater FM symptom severity and poorer QoL. Also, ADHD scores would mediate the relationship between depression severity and QoL.

## 1. Introduction

Attention-deficit/hyperactivity disorder (ADHD) is a childhood-onset neurodevelopmental disorder characterized by inattention, hyperactivity, and impulsivity. ADHD affects approximately 5.29% to 7.10% of children [1] and can persist into adulthood in up to 65% of cases [2]. The hypodopaminergic state seems to play a central role in ADHD. A great body of evidence demonstrates that people with ADHD tend to be at greater risk for other mental disorders [3] and metabolic conditions (such as migraine and obesity) [4]. The other clinical condition in individuals with ADHD is pain disorders. Individuals with ADHD are more sensitive to pain than healthy controls. Fibromyalgia (FM) is one of the most important pain disorders [5].

FM is a widespread musculoskeletal pain disorder accompanied by fatigue, stiffness, sleep disturbance, and cognitive impairment [6]. In addition to these medical conditions, FM patients often have other comorbidities (including irritable bowel syndrome, interstitial cystitis, and mood disorders) [7]. Thus, FM may impair quality of life (QoL) and has a large economic burden worldwide [8].It has been reported that FM affects 2.7% of the general population [9] and is seen in individuals aged 20 to 55 years, with a female to male ratio of 6:1 [10]. The etiology of FM has not been completely elucidated; however, several studies have proposed the role of genetic factors. Candidate genes relevant to the pathogenesis of FM include serotonergic, dopaminergic, and noradrenergic transporter polymorphisms of genes involved in pain transmission (such as the dopamine D4 receptor gene, catechol-O-methyltransferase, and serotonin transporter [11,12]. FM is defined as “pain amplification syndrome,” which contains increased spinal dorsal horn neuronal activity and diminished descending inhibition [13].

Psychiatric disorders generally accompany FM. Anxiety disorder and depression are among the most common psychiatric disorders [14]. These disorders have been related to a negative impact on fatigue, sleep, pain, physical function, and QoL in FM [15]. Also, FM patients exhibit cognitive disturbances including poor concentration, attention deficit, impaired short-term memory, forgetfulness, word-finding problems, inability to multitask, and fluency problems [16]. These cognitive disturbances called fibrofog can more affect patients than their widespread pain [17]. Although cognitive disturbances are universally experienced by FM patients, cognitive symptoms frequently remain underrecognized and undertreated, partly because the neuropsychological assessment of dyscognition is a complex process [18]. It has been reported that the prevalence of cognitive impairment is 2.5 times higher among FM patients compared with patients with other rheumatologic disorders and that 76.4%–82.5% of FM patients can suffer from cognitive difficulties [19]. Other studies showed that the rates of “concentration difficulties” and “failed memory”, which are the main symptoms of ADHD, were respectively 95% and 93% in FM patients [2,20].

In light of this information, some data make it necessary to evaluate the relationship between ADHD and FM. First, similar neurotransmitter pathways (norepinephrine and dopamine) has a crucial role in the etiology of both disorders. Restless legs syndrome, which is considered to be a dopamine dysregulation disorder [21], has been reported in up to 33% of ADHD patients [22] and up to 31% of FM patients [23]. Second, cognitive disturbances are a major clinical part of both disorders. Finally, case-control studies are reporting that ADHD medications are effective in both ADHD symptoms and FM-related pain [24]. Thus, we were aimed to determine the association between ADHD symptoms severity, FM severity, and QoL.

Hypothesis:

(1) ADHD symptoms are more common in patients with FM than in the control group.

(2) There was a negative correlation between the ADHD symptoms severity and QoL, and a positive correlation with the FM severity.

(3) ADHD scores would mediate the relationship between depression severity and quality of life.

## 2. Materials and methods

### 2.1. Study design and participants

We used observational cross-sectional survey design. In the study in which 153 participants were included; 113 were FM patients and 40 were the control group. The age range of the participants was 18–65. Education and Research Hospital Physical Medicine and Rehabilitation Clinic was held in Konya/ Turkey between March 2019 and March 2020. Data of participants were randomly 113 female patients with FM were collected consecutively from the physical medicine and rehabilitation unit. Both data collection and assessment procedures were done blindly. Participants who were newly diagnosed with FM were included in the study. The physician diagnosed the FM according to the 2016 criteria established by the American College of Rheumatology (ACR). Three conditions must be met in order to be diagnosed according to ACR: 1) having symptoms of similar severity for at least 3 months; 2) widespread pain index (WPI) ≥7 and symptom severity scale (SSS) score ≥5 or WPI of 4–6 and SSS score ≥9; 3) Generalized pain in at least 4 of the 5 regions. In addition, the presence of FM does not exclude the presence of other diseases. Those with medical conditions considered to contribute to FM symptomology (such as thyroid disorders, rheumatoid arthritis, systemic lupus erythematosus, inflammatory arthritis, sjögren’s syndrome, myositis, and vasculitis) were excluded from the study. Those who scored higher than the cut-off values for mental health screening tools applied to the participants by the physician and who had psychiatric complaints were referred to the psychiatrist in terms of the presence of ADHD and other psychiatric diagnoses. Those who had a psychiatric diagnosis other than ADHD were not included in the study. DSM-5 diagnostic criteria were used to diagnose ADHD. Each participant’s psychiatric/physical evaluation and psychometric tests were completed within an average of 90 min. The control group was composed of 40 age-matched patient relatives and hospital staff who were subjected to the same exclusion criteria as for the FM patients. There was no significant difference between the two groups in terms of marital status, income level, and educational level. Income level was classified into three groups as low, medium and high, according to the latest data from the relevant institutions in Turkey. A total of 217 patients with FM were evaluated for participation in the study. However, 104 of them were excluded from the study, 15 because of refusing participation and 89 because of not meeting the inclusion criteria. 

The sociodemographic data collection form, adult ADHD self-report scale (ASRS), fibromyalgia impact questionnaire (FIQ), World Health Organization quality of life scale-brief version (WHOQOL-BREF), and hospital anxiety and depression scale (HADS) were used to evaluate the study participants. Required permissions for this study were obtained from the Local Ethics Committee of Selcuk University.

### 2.2. Tools

#### 2.2.1. Adult ADHD self-report scale (ASRS)

It was developed for screening ADHD in adults by the World Health Organization [25]. It was designed to investigate the prevalence of ADHD in the general population and was recommended for use in primary screening clinical trials on ADHD. It is a short self-administered questionnaire that is based on existing symptoms, unlike tools that retrospectively define childhood ADHD. It is rated using a five-point Likert scale (never (0), rarely (1), sometimes (2), often (3), and very often (4) and has two subscales with each subscale consisting of nine questions (‘attention deficit’ and ‘hyperactivity/impulsivity’). These questions are created to determine how often each symptom happens within the last 6 months. It was reported that those with scores of 24 or higher from any of the two subscales had “a high probability of ADHD” (ADHD), those with scores of 17–23 had “likely ADHD”, and those with scores of 0–16 had no ADHD. The validity and reliability of the Turkish version of the scale were made by Doğan et al. [26]. The Cronbach’s alpha coefficient of the translated and original versions were 0.88 and 0.70.

#### 2.2.2. Fibromyalgia impact questionnaire (FIQ)

It is a widely used scale to assess clinical severity in FM patients. It is a Likert-type scale that evaluates 10 different areas in FM patients. These areas are; anxiety, depression, well-being, stiffness, fatigue, work difficulty, morning tiredness, physical functioning, missed days of work, and pain. The total score of the scale is between 0 and 100 and a higher score indicates a more negative impact. Scores of 70 and above indicate severe engagement. FIQ was developed by Burchardt et al. [27] and a validity and reliability study was conducted for Turkish FM patients in 2000 [28]. The Cronbach’s alpha coefficient of the translated and original versions were 0.72 and 0.95, respectively.

#### 2.2.3. Hospital anxiety and depression scale (HADS)

Anxiety and depression symptom levels of FM patients were evaluated with HADS. HADS-A subscale is used for anxiety symptoms and the HADS-D subscale is used for depression symptoms. The maximum score is 21 on both subscales, which are rated as a four-point Likert (0–3) and consist of 7 items. Higher scores show increased anxiety and depression symptom severity. The cutoff point was 7 for the depression subscale and 10 for the anxiety subscale. The Turkish validation and reliability of this scale, developed by Zigmond and Snaith [29], was made in 2003 [30]. The Cronbach’s alpha coefficient of the depression and anxiety subscales were 0.77 and 0.85, respectively.

#### 2.2.4. World Health Organization quality of life scale-brief version (WHOQOL-BREF)

It was developed as a 27-item short version of WHOQOL-100 to reduce the burden on participants in clinical trials. Turkish validity and reliability study of the scale was published by Eser et al. [31]. It evaluates the participants’ QoL in 5 different domains. These domains are; physical health, general health, psychological well-being, social relationships, and the environment. Each item is rated on a 5-point Likert scale. The scale, which is converted with a total score between 0 and 100 points, has no cutoff value. The Cronbach’s alpha coefficient were calculated as 0.73 for the environmental domain, as 0.66 for the psychological domain, as 0.73 for the national environmental domain, as 0.83 for the physical domain, and as 0.53 for the social domain, respectively.

### 2.3. Statistical analysis

IBM SPSS-23 program was used for data analysis (SPSS Inc; Chicago, IL, USA). Groups were compared using the independent samples t-test for parametric continuous variables (such as age and psychometric test scores) and the chi-square test for categorical variables (such as marital status, education, and ADHD rates). The relationships between clinical variables and test scores were analyzed using the Pearson correlation coefficients. The bootstrap method of Preacher and Hayes (2008) was used to determine the significance of the mediation effect in the study [32]. SPSS Macro Process program was used to calculate the Bootstrap method. Five thousand preloads were made in the calculation of the intermediary effect. Hayes’ (2013) Model 4 was used to identify the mediating role of ADHD scores in the effect of depression severity on quality of life [33]. Confidence intervals without zero indicate that the mediating effect is significant [32]. We also used the Sobel test to test significance of the mediation effect [34]. Probabilities less than 0.05 were used as the level of statistical significance in the analyzes.

## 3. Results 

While the FM group consisted of 113 (74%) patients, the control group consisted of 40 (26%) individuals. The mean age was 41.38 ± 9.86 years in the FM group and 40.52 ± 10.72 years in the control group. No significant differences were found in age, education level, and marital status between the two groups (Table 1). 

**Table 1:** Descriptive statistics of FM and control groups.

Participants	FM(n=113)	Control(n=40)	Statistical Analyses
Age	41.38± 9.86	40.52 ± 10.72	p = 0.63 t=0.48
Educational level of the Patients, (n%)			p = 0.84 x2=0.805
Primary school	11 (9.7%)	3 (7.5%)	
Secondary school	27 (23.9%)	12 (30.0%)	
High school	48 (42.5%)	16 (40.0%)	
University	27 (23.9%)	9 (22.5%)	
Marital Status, (n%)			p = 0.78 x2=0.740
Married	98 (86.7%)	34 (85.0%)	
Single	15 (13.3%)	6 (15.0%)	
Level of income (per month), (n%)			p = 0.50 x2=1.385
Low (<1000 $)	37 (32.7%)	16 (40.0%)	
Middle(1000$<, <2000$)	63 (55.8%)	18 (45.0%)	
High (>2000$)	13 (11.5%)	6 (15.0%)	
Work, (n%)			p = 0.89 x2=0.016
Work	55 (48.7%)	19 (47.5%)	
Not work	58 (51.3%)	21 (52.5%)	

ADHD was detected in 38.9% (n = 44) of the FM group and in 5% (n = 2) of the control group (p < 0.001). It was found that the FM group had significantly higher scores on the ASRS, all WHOQOL-BREF domains, HADS-A, and HADS-D than the control group (p < 0.05) (Table 2).

**Table 2:** Analysis of variables between FM and control groups for WHOQOL-BREF, ASRS, and HADS scores.

	FM	Controls	Statistical analyses
Participants screened	113	40	
WHOQOL-GH	39.00 ± 19.85	64.68 ± 13.54	p < 0.001 t = –7.574
WHOQOL-PH	44.82 ± 17.42	75.44 ± 12.81	p < 0.001 t = –10.173
WHOQOL-P	53.11 ± 17.26	68.54 ± 12.40	p < 0.001 t = –5.193
WHOQOL-E	60.54 ± 15.43	70.93 ± 15.52	p < 0.001 t = –3.656
WHOQOL- SR	53.77 ± 15.71	68.12 ± 16.44	p < 0.001 t = –4.504
HADS_A	10.55 ± 4.35	5.70 ± 2.52	p < 0.001 t = 6.664
HADS_D	8.16 ± 3.45	3.92 ± 2.84	p < 0.001 t = 6.980
ASRS TOTAL	25.60 ± 12.86	14.12 ± 5.74	p < 0.001 t = 5.445
ASRS ATTENTION	10.61 ± 4.99	5.70 ± 3.36	p < 0.001 t = 5.775
ASRS HYPERACTIVITY/IMPULSIVITY	14.90.13 ± 9.24	8.42 ± 3.85	p < 0.001 t = 4.293

The FM group was further divided into two subgroups as FM alone and comorbid FM/ADHD. There was a significant difference in FIQ scores and three WHOQOL-BREF domain scores (general health/ psychological health/physical health) between the FM alone and comorbid FM/ADHD groups (p < 0.05) (Table 3).

**Table 3:** Analysis of variables between alone FM and FM/ADHD groups for FIQ, WHOQOL-BREF, ASRS, and HADS scores.

	Alone FM	FM/ADHD	Statistical analyses
Participants screened, No. %)	69 (61.1%)	44 (38.9%)	
FIQ	58.28 ± 16.50	74.50 ± 16.28	p < 0.001 t = –5.124
WHOQOL -GH	33.12 ± 12.46	42.75 ± 21.00	p = 0.01 t = 2.577
WHOQOL PH	40.17 ± 16.97	47.78 ± 17.18	p = 0.02 t = 2.307
WHOQOL-P	46.96 ± 15.88	57.02 ± 17.07	p = 0.02 t = 3.137
WHOQOL-E	58.59 ± 15.73	61.78 ± 15.22	p = 0.28 t = –0.397
WHOQOL SR	53.18 ± 15.33	54.54 ± 21.07	p = 0.69 t = 1.070
HADS-A	9.82 ± 4.59	11.70 ± 3.70	p = 0.02 t = 2.278
HADS-D	7.73 ± 3.42	8.84 ± 3.42	p = 0.10 t = –1.668

In the analysis using the Pearson correlation coefficient, there was a high negative correlation between the ASRS total score and all other subscales of the WHOQOL-BREF (except for the social relationships domain score) and a high positive correlation with the FIQ, HADS-A, and HADS-D. The results of the Pearson correlation analysis are shown in Table 4.

**Table 4:** Pearson’s correlation coefficients for each pair of variables.

	1	2	3	4	5	6	7	8	9	10	11
1. Age											
2. WHOQOL-GH	–0.146										
3. WHOQOL-P	–0.166	0.618**									
4. WHOQOL-PH	–0.062	0.484**	0.593**								
5. WHOQOL-E	–0.013	0.280**	0.561**	0.478**							
6. WHOQOL-SR	–0.167	0.112	–0.453**	0.193*	0.370**						
7. HADS-A	–0.135	–0.353**	–0.471**	–0.366**	–0.273**	–0.057					
8. HADS-D	0.239*	–0.513**	–0.553**	–0.365**	-0.263**	–0.297**	0.367**				
9. ASRS-T	–0.037	–0.259**	–0.370**	–0.433**	-0.212*	–0.066	0.275**	0.246**			
10. ASRS-A	–0.058	–0.320**	–0.390**	–0.410**	–0.145	–0.134	0.316**	0.319**	0.816**		
11. ASRS-H/I	–0.015	–0.206*	–0.327**	–0.383**	–0.216*	–0.036	–0.216*	0.184	–0.288**	0.947**	
12.FIQ	0.162	–0.582**	–0.524**	–0.616**	-0.293**	–0.110	0.410**	0.431**	0.557**	0.508**	0.508**

Hypothesis 3 predicted that ADHD scores would mediate the relationship between depression severity and QoL. To test this hypothesis, the SPSS process mediation test was performed to evaluate the mediation effect of ASRS-TOTAL between HADS-D and the WHOQOL-PH. Figure A shows that the total effect of HADS-D on WHOQOL -PH was significant (β = 0.18, t = 2.63, p = 0.010). The 95% bias-corrected confidence intervals did not contain zero (32), which revealed that ASRS-TOTAL was a mediator in the relationship between HADS-D and the WHOQOL-PH, [β = –0.82, % 95 CI (–1.7513, –0.0430)]. This indicates partial mediation. Partial mediation indicates that some of the relationships between HADS-D on WHOQOL-PH occurs directly, while others are indirectly based on ASRS-TOTAL (32). The results of the mediation analyses are presented in Figure B. Results of the Sobel test indicate ADHD scores significantly mediated the relationship between depression severity and QoL (Z = –2.33, p < 0.05).

**Figure:**
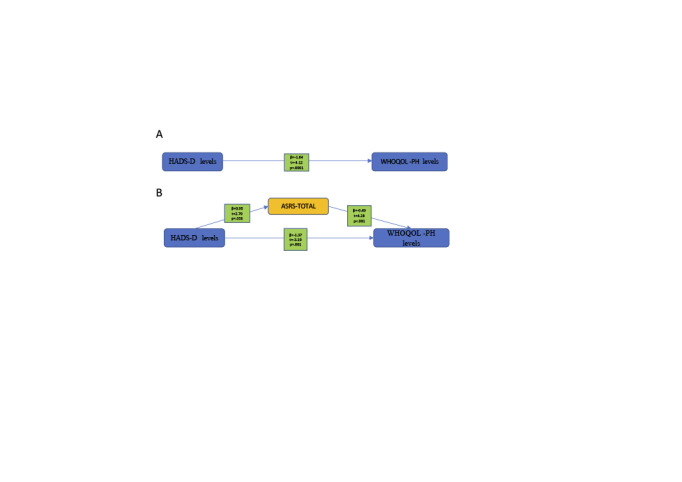
Mediator effects of ASRS. WHOQOL-PH: World Health Organization questionnaire on quality of life-physical health, HADS-D: hospital anxiety and depression scale-depression, ASRS: adult ADHD self-report scale

## 4. Discussion

FM continues a complex pain disorder due to multiple comorbidities. In our study, the effect mechanism of ADHD symptoms presence on QoL in patients with FM was evaluated. The possible mediating effect of ADHD scores in the relationship between depression symptoms severity and QoL has never been investigated before.

The possible overlap of FM with ADHD is underestimated, and thus the presence of ADHD in FM patients may be underreported. Indeed, studies have shown that the frequency of FM appears to be higher among adults with ADHD compared to controls or even patients with cognitive complaints without ADHD. Conversely, adult ADHD is more commonly seen in FM [35]. Persistent pain poses a definite effect on attention. Moore et al. (2012) demonstrated that it influenced complex attention performances (such as attention span, attention shifting, and divided attention) rather than simpler attention tasks [36]. Van Rensburg et al. found that 44.7% of patients with FM were diagnosed with comorbid adult ADHD according to the ASRS v1.1 [37]. Another study showed that 25% of FM patients had co-occurring ADHD [38]. Our study found that the rate of ADHD in patients with FM was 38.9%. 

This higher level of coexistence requires a common pathogenic perspective for both conditions. The common comorbidity of FM and adult ADHD can produce various therapeutic effects, as defects in dopaminergic circuits have been implicated in both cases. For instance, FM is associated with reduced repetition of the dopamine D4 receptor gene (DRD4), which is normally characterized by seven polymorphic repeats [39]. These seven repetitive variations are related to thinner right orbitofrontal, inferior prefrontal, and posterior parietal cortex in ADHD [40]. FM is characterized by decreased activity of the descending pain inhibitory pathways and low levels of dopamine, serotonin, norepinephrine, and endogenous opioids [41]. ADHD is characterized by abnormal neurodevelopmental processes that affect attention, emotion, and somatosensory functions, especially in frontostriatal, frontoparietotemporal, frontocerebellar, and frontolimbic regions. Both dopaminergic and noradrenergic systems are responsible for the greatest impairment in ADHD [42]. The other problematic area for both cases is emotional regulation. Deficits in recognition, regulation, and expression of emotions are one of the main problems in both ADHD and FM [43]. For the reasons mentioned above, the common etiologic factors that may be effective in the etiology and symptom severity of FM should be evaluated in each patient. 

Another perspective in the relationship between ADHD and FM is that pain affects cognitive structures by disrupting working memory [44] and can be the best indicator for objective results of cognitive function test [45]. Pain is considered a high-priority signal. It is thought that pain slows, stops, or impairs behavior when high cognitive demand is present [46]. Cognitive impairment in FM cannot be fully explained by pain. Other underlying factors contain anxiety, depression, sleep, and fatigue [47]. Therefore, early intervention for problems other than cognitive deficits is very important for preventing cognitive disturbances in FM. 

The fact that ADHD symptoms increase the severity of FM symptoms and worsen QoL in patients with FM is one of the important data from our study. In a recent study examining this relationship, ADHD symptoms were indicated to be associated with poor functionality in patients with FM. This relationship was even more pronounced in the presence of depression and anxiety symptoms [48]. In another study, the presence of comorbid ADHD was found to be associated with increased severity of FM symptoms and increased impairment in functionality [49]. Although some observations have previously been conducted for the link between adult ADHD and FM based on shared cognitive deficits, a small number of studies have been performed on the level of QoL and ADHD severity in FM patients. In addition to the fact that ADHD symptoms are more frequent in FM patients, our study demonstrated that the increased severity of ADHD symptoms would pose a negative impact on multiple domains of the QoL. Our study also showed that the general health, psychological health, physical health, environment domains of the WHOQOL-BREF strongly correlated with ADHD symptom severity. Until recently, cognitive dysfunction experienced by FM patients has been largely ignored due to the lack of diagnostic criteria. Cognitive dysfunction, such as memory and attention, can sometimes dramatically change the lives of FM patients. Patients with FM often report declines in cognitive function, memory, and mental alertness as well as forgetfulness that have been called “fibro fog”, which can be more disruptive than pain. Therefore, it should be one of the most important parts of clinical assessment in FM patients [19].

In the present study, it is revealed that ADHD scores of the patients with FM have a partial mediating effect in the relationship depression severity and physical quality of life. It has been shown in many studies that FM patients show an increased rate of anxiety and depressive symptoms besides their cognitive symptoms [50]. In fact, depressive disorder is the most common comorbidity with a prevalence of 20%–80% [51]. It has been shown that somatic symptom/pain is more common when it accompanies depression in FM patients, related to the “pain relief hypothesis” [15,52]. The presence of these comorbidities is important in that they seriously affect the QoL [53], apart from making treatment difficult. Emotion regulation problems, which are key components in ADHD, may cause sensitivity to depressive symptoms [54]. In addition, due to the cognitive and emotional symptoms of ADHD in FM patients, dysfunctional coping mechanisms may contribute to the development of depressive symptoms. While evaluating the psychical QoL, which is an important target in the FM management process, consideration of ADHD can make a significant contribution to treatment and prognosis. Our findings revealed that depression has an effect on QoL through ADHD symptoms. It can be interpreted that intervention in emotion regulation deficits/emotional responsiveness and other features mediating ADHD-related depression may have an effect on QoL in patients with FM.

The present study has several limitations. First, it was not possible to exclude the influence of confounding factors such as physical exercise, sleep habits, and eating behavior. Second, our study included a relatively small number of subjects. Another limitation of our study is that all patients were not evaluated with a structured psychiatric interview. Furthermore, the absence of male FM patients in our study may limit the generalizability of the results. Finally, the relationship between FM and hyperactivity/impulsivity were not adequately addressed, but the attention and related factors were mainly evaluated. 

In conclusion, our findings indicated that in FM patients with ADHD, FM symptom severity is higher and the QoL is worse. Also, the partial mediating effect of ADHD symptom severity in the relationship between depression severity and physical QoL is an important finding. Thus, in FM patients may not respond adequately to standard treatment if their ADHD symptoms are not taken into consideration.

## Informed consent

The study protocol received institutional review board approval. All participants provided informed consent in the format required by the relevant authorities.
